# PACAP Modulates the Autophagy Process in an In Vitro Model of Amyotrophic Lateral Sclerosis

**DOI:** 10.3390/ijms21082943

**Published:** 2020-04-22

**Authors:** Agata Grazia D’Amico, Grazia Maugeri, Salvatore Saccone, Concetta Federico, Sebastiano Cavallaro, Dora Reglodi, Velia D’Agata

**Affiliations:** 1 Department of Human Science and Promotion of quality of Life, San Raffaele Open University of Rome, Via di Val Cannuta, 247, 00166 Roma, Italy; agata.damico@uniroma5.it; 2 Section of Human Anatomy and Histology, Department of Biomedical and Biotechnological Sciences, University of Catania, Via S. Sofia, 87, 95123 Catania, Italy; grazia.maugeri@libero.it; 3 Section of Animal Biology, Department of Biological, Geological and Environmental Sciences, University of Catania, 95123 Catania, Italy; saccosal@unict.it (S.S.); federico@unict.it (C.F.); 4 Institute for Biomedical Research and Innovation, Italian National Research Council, 95123 Catania, Italy; sebastiano.cavallaro@cnr.it; 5 Department of Anatomy, MTA-PTE PACAP Research Group, University of Pecs Medical School, 7622 Pécs, Hungary; dora.reglodi@aok.pte.hu

**Keywords:** PACAP, ALS, autophagy process, hypoxia condition

## Abstract

Amyotrophic lateral sclerosis (ALS) is a progressive neurodegenerative disease of complex etiology leading to motor neuron degeneration. Many gene alterations cause this pathology, including mutation in Cu, Zn superoxide dismutase (SOD1), which leads to its gain of function. Mutant SOD1 proteins are prone to aberrant misfolding and create aggregates that impair autophagy. The hypoxic stress is strictly linked to the disease progression since it induces uncontrolled autophagy activation and the consequent high rates of cell death. Previously, we showed that pituitary adenylate cyclase-activating polypeptide (PACAP) exerts neurotrophic activity in cultured mSOD1 motor neurons exposed to serum deprivation. To date, no studies have examined whether the protective effect of PACAP on mSOD1 cells exposed to hypoxic insult is mediated through the regulation of the autophagy process. In the present study, we used the neuroblastoma-spinal cord-34 (NSC-34) cell line, stably expressing human wild type or mutant SOD1 G93A, to represent a well characterized in vitro model of a familial form of ALS. These cells were exposed to 100-µM desferrioxamine mesylate salt for 24h, to mimic the hypoxic stress affecting motor neurons during the disease progression. Our results showed that PACAP treatment significantly reduced cell death and hypoxia-induced mSOD1 accumulation by modulating the autophagy process in G93A motor neurons, as revealed by the decreased LC3II and the increased p62 levels, two autophagy indicators. These results were also confirmed by evaluating the vacuole formation detected through light chain 3 (LC3) immunofluorescence. Furthermore, the PACAP effects on autophagy seem to be mediated through the activation of the MAPK/ERK signaling pathway. Overall, our data demonstrated that PACAP exerts an ameliorative effect on the mSOD1 motor neuron viability by modulating a hypoxia-induced autophagy process through activation of MAPK/ERK signaling cascade.

## 1. Introduction

Amyotrophic lateral sclerosis (ALS) is a progressive neurodegenerative disease of complex etiology, characterized by a motor neuron degeneration in the spinal ventral horn, cerebral cortex and brainstem nuclei leading to muscle weakness [1,2]. 

It is classified in two different forms: a sporadic (sALS) and a familial (fALS) form [3]. The latter is due to different gene mutations, including the Cu, Zn superoxide dismutase (SOD1) gene [4,5]. Although the pathogenetic mechanism involved in ALS caused by SOD1 mutation is not yet well understood, it has been suggested that misfolding or the aggregation of the encoded protein represented a key event in disease development [6]. 

Since the cytoplasm of the motor neurons in ALS patients and mutant SOD1 (mSOD1) animals contains characteristic aggregates including autophagy-related protein LC3II, it has been hypothesized that the aberrant induction of the autophagy process was implicated in disease pathogenesis [7]. It has also been demonstrated that the overexpression of mSOD1 induced an increase in the autophagic activity in an in vitro model of ALS [8]. 

Considering the prominent role of autophagy in cleaning and maintaining the regular turnover of cellular cytoplasmic components, it has been suggested that its modulation may be beneficial in ALS-affected motor neurons. Therefore, the identification of molecules interfering with specific autophagy steps may allow the discovery of new therapeutic targets [9,10]. 

To better characterize the molecular mechanism underlying ALS, in the last decade many in vitro and in vivo models with mSOD1 have been developed. In particular, the transgenic animal mSOD1 G93A is the most common model used to study the etiopathogenesis of ALS since they show some clinical signs characteristic of the disease [11,12]. 

In animal models, as well as in sALS patients, the alteration of the autophagy process has been linked to impairment in the hypoxic signaling pathway [13,14]. In physiological conditions, cellular responses to hypoxia are represented by the activation of the autophagy followed by changes in energy metabolism [15]. This insult is frequently related to the onset of different degenerative diseases, including ALS, since low oxygen supply induces microenvironmental changes rendering motor neurons more vulnerable to cell death [13,16]. In particular, in mSOD1 G93A mice, hypoxia was considered to be one of the causes of motor neuron death [17]. In this regard, Cimini et al., (2014) demonstrated that G93ASOD1 cells are more susceptible to hypoxic stress inducing the dysregulation of autophagy, a mechanism involved in misfolded mSOD1 protein clearance [18]. Furthermore, a recent epidemiological study highlighted that the intermittent exposure to hypoxic insult, as occurs for firefighters, represents an occupational risk for ALS [19]. 

To date, many chemical agents or neurotrophic factors have been tested in clinical trials for ALS; however, only two molecules were approved by the Food and Drug Administration (FDA) to delay disease progression: riluzole and edaravone, antiexcitotoxic and antioxidant substances, respectively [20]. 

In recent years, the wide genomic expression analysis performed by DNA microarrays has allowed for the identification of deregulated genes in ALS and new potential drug targets for the treatment of the disease [21]. The analysis of the genomic profiles of 41 motor cortex samples identified the deregulation of the pituitary adenylate cyclase-activating polypeptide (PACAP) gene in a subgroup of sporadic ALS patients [21]. This peptide has neuroprotective and neurotrophic effects in different models of neurodegeneration through binding to three different G protein-coupled receptors known as PAC1, VPAC1 and VPAC2 [22,23,24,25,26,27,28]. Its exogenous administration ameliorated the cognitive performance in an animal model of Alzheimer’s disease as well as motor function in a mouse model of Parkinson’s disease [29,30]. Furthermore, it has been demonstrated that it is significantly upregulated in the motor or sensory neurons following different types of insults, such as inflammation and peripheral nerve injury [31,32,33,34]. The pro-survival effect of this peptide has also been demonstrated in primary culture motor neurons and during ALS progression [35,36]. More recently, we demonstrated its protective effect in an in vitro model of ALS, where it counteracted apoptotic death in human-induced pluripotent stem cells (iPSC)-derived motor neurons [37]. Furthermore, we showed that the peptide was able to prevent cell degeneration following the growth factor deprivation in mSOD1 G93A motor neurons by increasing EGFR tyrosine phosphorylation through protein kinase A (PKA) activation [38]. 

To better characterize the effect of PACAP in ALS motor neuron degeneration, in the present study we investigated its involvement in the autophagy process by using an in vitro model of neuroblastoma-spinal cord-34 (NSC-34) cells stably bearing a human Cu/Zn superoxide dismutase1 G93A mutation (G93A) exposed to hypoxic insult.

The results suggest that PACAP prevented mSOD1 motor neuron death through the regulation of cellular homeostasis by modulating the autophagy processes mediated by MAPK/ERK signaling cascade activation.

## 2. Results

### 2.1. PACAP Effect on Human Wild-Type-SOD1 (WT) and G93A Cell Viability after Hypoxic Insult

**Figure 1:**
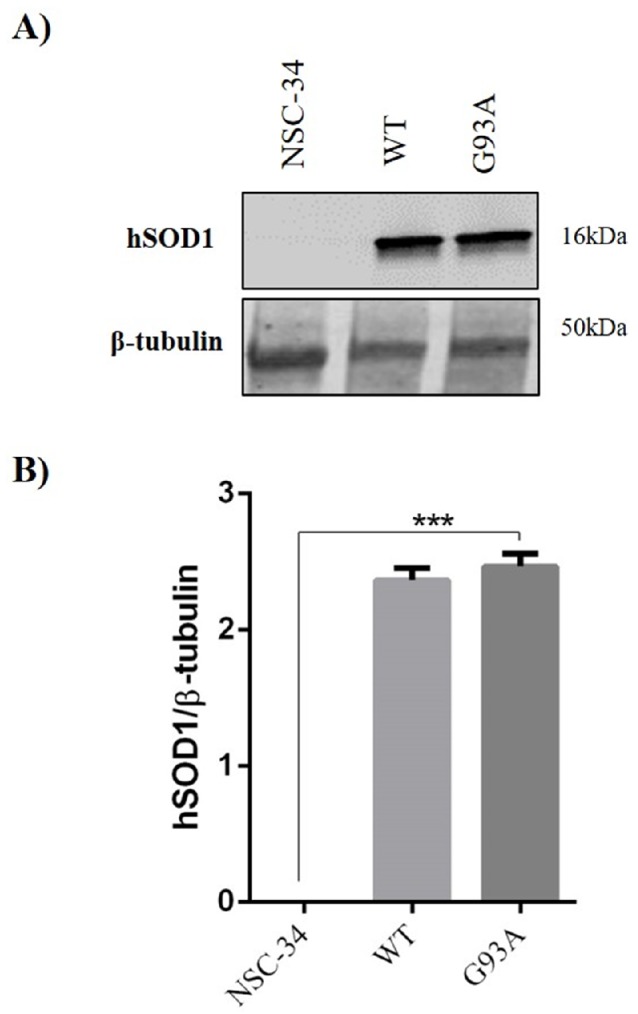
The human Cu/Zn superoxide dismutase1 (hSOD1) expression in neuroblastoma-spinal cord-34 (NSC-34), human wild-type-SOD1 (WT) and G93A cells following doxycycline induction. (**A**) Representative immunoblots of the signals detected for hSOD1 expression in NSC-34, WT and G93A cell homogenates after induction with 2 µg/mL doxycycline. (**B**) The bar graph shows the results of three independent experiments. Relative band density was quantified by using the ImageJ software and the protein levels are expressed as the arbitrary units obtained after normalization to β-tubulin, which was used as a loading control. Data are expressed as a mean ± SEM (*** *p* < 0.01 vs. NSC-34, as determined by a one-way ANOVA followed by Tukey’s post-hoc test).

To confirm the validity of the in vitro model used, we carried out a preliminary experiment to evaluate the human Cu/Zn superoxide dismutase1 (hSOD1) expression following doxycycline cell treatment. To conduce this analysis, we used the monoclonal antibody anti-superoxide dismutase 1 that specifically recognizes human SOD1. As previously described by Arciello et al. (2010) [39], hSOD1 is undetectable in NSC-34 cells whereas its expression increases significantly in both WT and G93A cells (Figure 1). 

**Figure 2:**
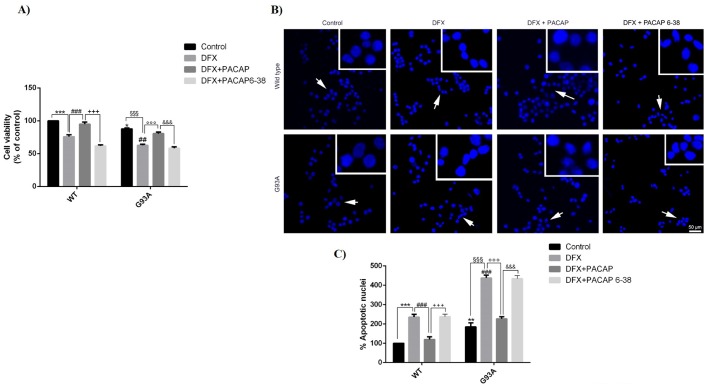
Pituitary adenylate cyclase-activating polypeptide (PACAP) effect on the WT and G93A cell viability exposed to hypoxia for 24h. (**A**) Cell viability was determined in WT and G93A cultured for 24h in desferrioxamine mesylate salt (DFX) alone or in combination with PACAP or PACAP 6-38 by using an MTT assay. Results are representative of three independent experiments and the bar graph shows the values expressed as a percentage of the control (* *p* < 0.05 or *** *p* < 0.001 vs. WT control, ^##^ *p* < 0.01 or ^###^ *p* < 0.001 vs. WT DFX, ^+++^ *p* < 0.001 vs. WT DFX + PACAP, ^§§§^ *p* < 0.001 vs. G93A control, ^°°°^ *p* < 0.001 vs. G93A DFX and ^&&&^ *p* < 0.001 vs. G93A DFX + PACAP as determined by a one-way ANOVA followed by Tukey’s post-hoc test). (**B**) The microphotographs show cells stained with the fluorescent nuclear dye Hoechst 33342. Scale bar = 50 μm. (**C**) The bar graph represents the mean ± SEM of apoptotic nuclei percentages calculated by counting seven fields per dish with a fixed pattern. Results are representative of three independent biological replicates. (** *p* < 0.01 or *** *p* < 0.001 vs. WT control, ^###^ *p* < 0.001 vs. WT DFX, ^+++^ *p*< 0.001 vs. WT DFX + PACAP, ^§§§^ *p* < 0.001 vs. G93A control, ^°°°^ *p* < 0.001 vs. G93A DFX and ^&&&^ *p* < 0.001 vs. G93A DFX + PACAP as determined by a one-way ANOVA followed by Tukey’s post-hoc test).

The increased susceptibility of G93ASOD1 motor neuronal cells to a hypoxic environment has previously been reported [18]. To determine the effect of PACAP against hypoxia-induced cell death, we performed an MTT assay in WT and G93A cells exposed for 24 h to desferrioxamine mesylate salt (DFX) alone or in combination with PACAP (DFX + PACAP) or PACAP 6-38 (DFX + PACAP 6-38), a specific inhibitor of the PAC1 receptor. In agreement with our previous paper, doxycycline-induced human SOD1 expression in G93A cells resulted in a reduction of their viability as compared to the WT group (Figure 2, * *p* < 0.05 vs. WT control) [38]. The exposure to the hypoxic-mimetic agent significantly decreased cell viability in the WT and G93A cell lines as compared to the respective control groups (Figure 2A, *** *p* < 0.001 vs. WT control, ^§§§^ *p* < 0.001 vs. G93A control). However, the percentage of viable cells was significantly lower in the G93A cells compared to the WT cells (Figure 2A, ^##^ *p* < 0.01 vs. WT DFX), confirming that the SOD1 mutation rendered motor neurons more susceptible to this insult. The PACAP exogenous administration significantly increased cell survival in both the WT and G93A cells (Figure 2A, ^###^ *p* < 0.001 vs. WT DFX and ^°°°^ *p* < 0.001 vs. G93A DFX) and its effect was inhibited by the treatment with PACAP 6-38, a PAC1 receptor inhibitor (^+++^ *p* < 0.001 vs. WT DFX + PACAP and ^&&&^ *p* < 0.001 vs. G93A DFX + PACAP), suggesting that PACAP acted through binding to the PAC1 receptor.

The peptide’s protective activity was also confirmed by analyzing apoptotic cell death through a Hoescht 33342 staining assay. As shown in Figure 2B,C, the treatment with DFX significantly increased the percentage of apoptotic nuclei in the WT and G93A cells (** *p* < 0.01 or *** *p* < 0.001 vs. WT control and ^§§§^ *p* < 0.001 vs. G93A control); however, the death rate of G93A was higher than that for the WT cells (^###^ *p* < 0.001 vs. WT DFX). PACAP co-treatment significantly reduced apoptosis in both groups (Figure 2B,C, ^###^ *p* < 0.001 vs. WT DFX and ^°°°^ *p* < 0.001 vs. G93A DFX) and this effect was reverted by the PAC1 receptor inhibitor (^+++^ *p* < 0.001 vs. WT DFX + PACAP and ^&&&^ *p* < 0.001 vs. G93A DFX + PACAP).

**Figure 3:**
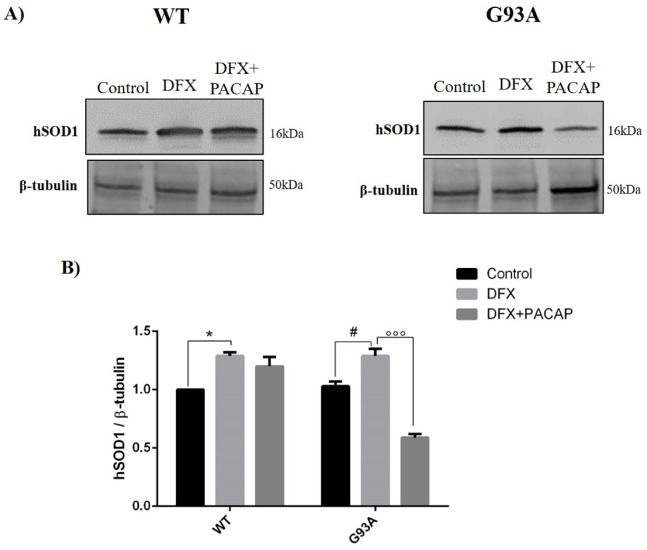
PACAP interferes with the hSOD1 expression in the WT and G93A cells under hypoxia. (**A**) Representative immunoblots of the signals detected for the hSOD1 expression on the WT and G93A cell homogenates cultured for 24 h in desferrioxamine mesylate salt (DFX) alone or in combination with PACAP (DFX + PACAP). (**B**) The bar graph shows the results of three independent experiments. Relative band density was quantified by using the ImageJ software and the protein levels are normalized to β-tubulin, which was used as a loading control. Data are expressed as a mean ± SEM of values normalized to the WT/control data set to 1.0 (* *p* < 0.05 vs. WT control, ^#^ *p* < 0.05 G93A control and ^°°°^ *p* < 0.001 vs. G93A DFX, as determined by a one-way ANOVA followed by Tukey’s post-hoc test).

Furthermore, we assessed the hSOD1 levels to evaluate whether hypoxic stress affected its expression. As shown in Figure 3, an increased accumulation of hSOD1 protein was revealed in DFX-treated groups as compared to the controls (* *p* < 0.05 vs. WT or G93A control), while PACAP administration significantly decreased the hSOD1 levels exclusively in G93A DFX-exposed cells (^###^ *p* < 0.001 vs. G93A DFX). 

### 2.2. PACAP Treatment Modulates DFX-Induced Autophagy in WT and G93A Cells

To investigate the involvement of PACAP in the modulation of hypoxia-induced autophagy, we examined the expression of two proteins linked to the autophagy process, LC3I-II and p62 in both WT and G93A cells cultured in a DFX-conditioned medium for 24 h. In particular, microtubule-associated protein1 light chain 3 (LC3I) was involved in autophagosome membrane formation leading to misfolded-protein degradation. In the initial step of autophagy, during the phagophore biogenesis, LC3I was converted to the LC3II form and then phagophores recruited the polyubiquitin-binding protein P62, which interacted with the misfolded mSOD1 proteins promoting its association to LC3II and leading to its degradation. During the autophagy process, p62 expression was inversely related to LC3II levels [40]. 

**Figure 4:**
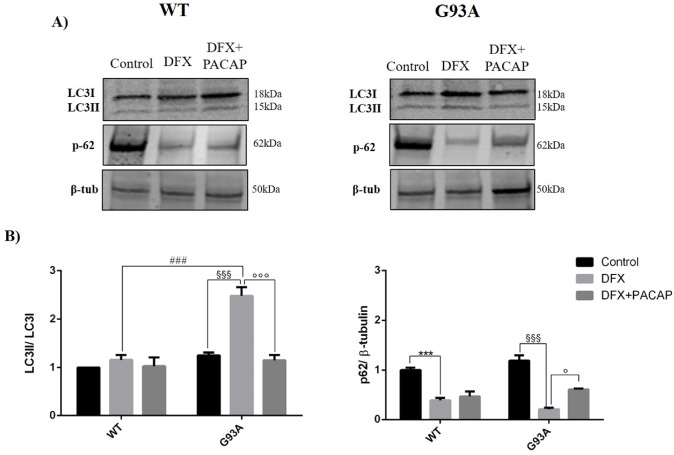
PACAP treatment modulates the light chain 3 (LC3) and p62 expression levels in the WT and G93A cells under hypoxia. (**A**) Representative immunoblots of the signals detected by the LC3I-LC3II and p62 antibodies on the WT and G93A cell homogenates cultured for 24 h in desferrioxamine mesylate salt (DFX) alone or in combination with PACAP (DFX + PACAP). (**B**) The bar graphs show the results of three independent experiments. Relative band density was quantified by the ImageJ software and the protein levels are normalized to LC3I or to β-tubulin, which was used as a loading control. Data are expressed as a mean ± SEM of values normalized to the WT/control data set to 1.0 (*** *p* < 0.001 vs. WT control, ^###^ *p* < 0.001 vs. WT DFX, ^§§§^ *p* < 0.001 vs. G93A control and ^°^ *p* < 0.05 or ^°°°^ *p* < 0.001 vs. G93A DFX as determined by a one-way ANOVA followed by Tukey’s post-hoc test).

As shown in Figure 4, the expression of LC3II was significantly increased in the DFX-treated G93A cells as compared to the controls as well as to the DFX-treated WT group (^§§§^ *p* < 0.001 vs. G93A control; ^###^ *p* < 0.001 vs. WT DFX). However, its level was significantly reduced following the PACAP treatment (^°°°^ *p* < 0.001 vs. G93A DFX). The peptide effect on LC3II levels was evidently linked to the hSOD1 mutation since the PACAP administration in WT cells did not affect its expression. 

As mentioned above, the LC3II level was inversely related to the p62 expression. Thus, our results showed that the p62 levels were significantly reduced after the DFX treatment in both the WT and G93A cells as compared to the controls (*** *p* < 0.001 vs. WT control, ^§§§^ *p* < 0.001 vs. G93A control) and the PACAP administration significantly increased its expression only in G93A cells (^°^ *p* < 0.05 vs. G93A DFX).

**Figure 5:**
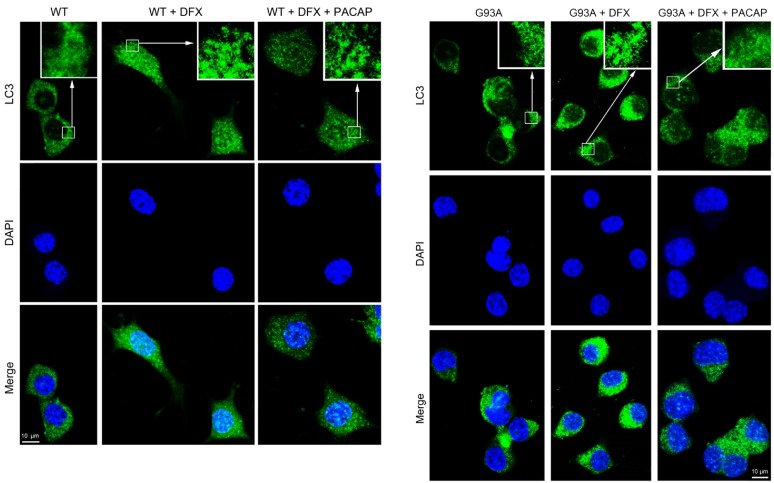
Immunosignal of LC3 protein in the WT and G93A cells exposed for 24h to hypoxia and treated with PACAP. The photomicrographs show the distribution of the LC3 protein in the WT and G93A cultured for 24 h with desferrioxamine mesylate salt (DFX) alone or in combination with PACAP (DFX + PACAP). The cytoplasmic immunofluorescent dots revealed the localization of the LC3 antibody in the autophagic vacuoles. The signal was detected using mouse anti-LC3 primary antibody revealed with the Alexa Fluor 488 secondary antibodies (green fluorescence). Cell nuclei were stained with diamidino-2-phenylindole, DAPI (blue fluorescence). The photomicrographs are representative results taken from different fields in randomly selected slides and scanned by confocal laser scanning microscopy (CLSM). Scale bar (10 µm).

Since the LC3 protein is a marker of autophagosome vacuoles [41,42], we performed immunofluorescence analysis. LC3 immunodetection revealed an increased number of bright puncta in the cytoplasm of the DFX-treated cells as compared to the controls. The exogenous PACAP administration reduced the autopathic vacuole formation in the G93A cells as revealed by the decreased bright dots in their cytoplasm (Figure 5).

### 2.3. PACAP Modulates Autophagy in WT and G93A Cells Exposed to Hypoxia through Activation of the MAPK/ERK Pathway

To study the signaling cascade underlying the PACAP effects on motor neurons exposed to hypoxia, we analyzed the survival pathway MAPK/ERK by assessing ERK phosphorylation in DFX-treated WT and G93A cells.

**Figure 6:**
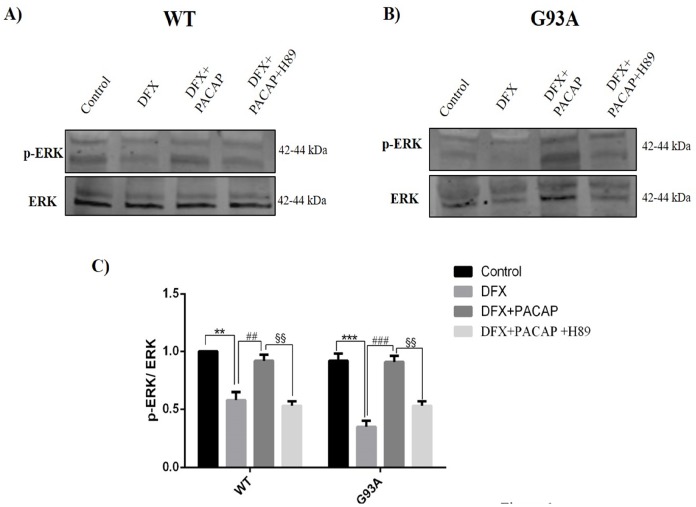
Effect of PACAP on ERK phosphorylation in the WT and G93A cells 24h after hypoxia exposure. (**A**,**B**) Representative immunoblots of the signals detected by the p-ERK/ERK antibody on the WT and G93A cell homogenates cultured for 24h in desferrioxamine mesylate salt (DFX) alone or in combination with PACAP (DFX + PACAP) or PACAP + H89 (DFX + PACAP + H89). (**C**) The bar graph show the results of three independent experiments. The ImageJ software was used to quantify the relative band density of the p-ERK/ERK protein levels. Data are expressed as a mean ± SEM of values normalized to the WT/control data set to 1.0 (** *p* < 0.01 or *** *p* < 0.001 vs. WT or G93A control, ^##^ *p* < 0.01 or ^###^ *p* < 0.001 vs. WT DFX and G93A DFX and ^§§^ *p* < 0.01 vs. WT and G93A DFX + PACAP as determined by a one-way ANOVA followed by Tukey’s post-hoc test).

As shown in Figure 6, p-ERK levels were significantly downregulated in the WT and G93A cells following DFX exposure with respect to the controls (** *p* < 0.01 or *** *p* < 0.001 vs. WT or G93A control). The PACAP treatment significantly enhanced its expression as compared to the DFX groups (^##^ *p* < 0.01 or ^###^ *p* < 0.001 vs. WT or G93A DFX). At 30 min with H89 co-treatment, an upstream MAPK/ERK pathway inhibitor, the expression levels of p-ERK were significantly reduced (^§§^ *p* < 0.01 vs. WT or G93A DFX + PACAP), suggesting that PACAP acts through the MAPK/ERK signaling cascade. 

**Figure 7:**
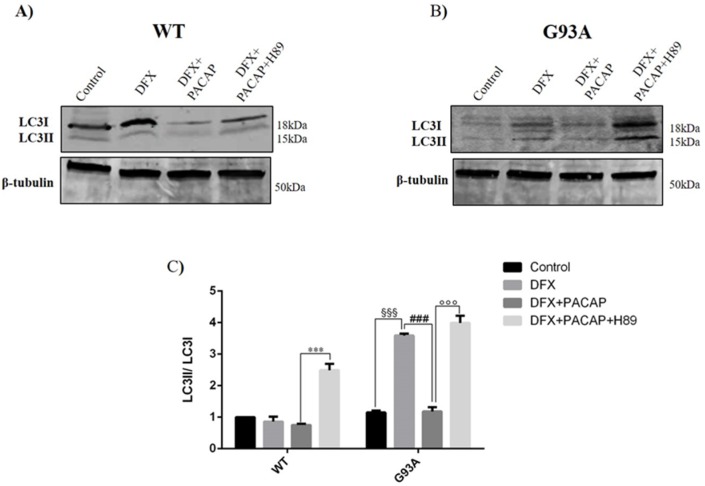
PACAP affects the LC3II expression levels in the WT and G93A cells through the activation of MAPK/ERK signaling. (**A**,**B**) Representative immunoblots of the signals detected by the LC3I-LC3II antibody on the WT and G93A cell homogenates cultured for 24 h in desferrioxamine mesylate salt (DFX) alone or in combination with PACAP (DFX + PACAP) or PACAP + H89 (DFX + PACAP + H89). (**C**) The bar graph shows the results of three independent experiments. Relative band density was quantified by the ImageJ software and the protein levels are normalized to LC3I. β-tubulin was used as a loading control. Data are expressed as a mean ± SEM of values normalized to the WT/control data set to 1.0 (^§§§^ *p* < 0.001 vs. G93A control, ^###^ *p* < 0.001 vs. G93A DFX, *** *p* < 0.001 vs. WT DFX + PACAP or ^°°°^ *p* < 0.001 vs. G93A DFX + PACAP as determined by one-way a ANOVA followed by Tukey’s post-hoc test).

To investigate whether the modulatory effect of PACAP on autophagy was mediated by the induction of the MAPK/ERK signaling pathway, we analyzed the LC3II expression in the DFX-treated WT and G93A cells following the peptide treatment alone (DFX + PACAP) or in co-treatment with H89 (DFX + PACAP + H89). As previously shown (Figure 4), the expression of LC3II was significantly increased in G93A DFX-treated respect to control (^§§§^ *p* < 0.001 vs. G93A control) and its expression was decreased in the G93A cells treated with DFX + PACAP as compared to the DFX-treated group (Figure 7C, ^###^ *p* < 0.001 vs. G93A DFX). The co-administration of H89 in G93A cells exposed to hypoxia significantly increased the LC3II levels with respect to the DFX + PACAP treated group (Figure 7C, ^°°°^ *p* < 0.001 vs. G93A DFX + PACAP), suggesting that PACAP-modulated autophagy was mediated through the MAPK/ERK signaling cascade activation. 

## 3. Discussion 

The typical degeneration of motor neurons in ALS is associated with the accumulation of abnormal protein aggregates [43]. To maintain cellular homeostasis, the protein aggregates are usually degraded through the autophagy–lysosome process [44]. During this process, the aggregate proteins are internalized in double-membrane vesicles known as autophagosomes that bind to lysosome membranes to degrade and recycle their content. Four steps characterize the autophagy process: the induction regulated by mTor; the autophagosome formation mediated by p62 and LC3II protein interaction; the fusion of the autophagosome with lysosome and the degradation of autophagic bodies inside the lysosome. This complex mechanism can be dysregulated by various stress-inducing insults contributing to the pathology development.

Mutant SOD1 proteins are prone to aberrant misfolding and impair some steps of the autophagy process leading to the hyperactivation of this biological event [2]. In this regard, it has been largely demonstrated that autophagy dysregulation linked to SOD1 mutation is strictly associated with ALS [7,45,46]. Nevertheless, the exact mechanism mediating this event is still not clearly understood, and autophagy modulation is considered a fundamental means to therapeutic approaches in ALS treatment. 

Hypoxia is a risk factor in neurodegenerative diseases including ALS [13,14]. The low oxygen tension induces the activation of several genes interfering with mitochondrial respiration and the production of reactive oxygen species (ROS). To counteract free radical production, changes occur in the cellular metabolism leading to the activation of the detoxifying system as well as scavengers and enzymes, including SOD1.

Here, we demonstrated that the exposure to hypoxia induces cell death and hSOD1 overexpression in both WT and mSOD1 G93A motor neurons, which represents a widely used in vitro fALS model [47]. However, this event seems to be related to the impairment of autophagic processes affecting only mutant SOD1 cells.

Previously, it has been demonstrated that mSOD1 encoded misfolded proteins that interacted with the autophagy adaptor p62 which enhanced the association between LC3II and mSOD1, leading to increase an autophagosome formation [48,49]. On the other hand, mSOD1-induced dysfunctions in autophagosome retrograde transport to the lysosome contributing to a failure of late-stage fusion steps. Overall, these events impair autophagy processes and lead to a SOD1 insoluble aggregate accumulation in motor neurons and consequently result in their death [2]. 

In line with these findings, we observed that hypoxia exposure resulted in a significant upregulation of the LC3II cytoplasmic expression in mSOD1 G93A motor neurons. Considering that increased LC3II levels are associated with autophagosome formation in early stages or with impairment during the degradation phase in later steps of autophagy, we also analyzed p62 expression since it bound LC3II during the autophagosome formation step.

Our results showed that increases in LC3II were related to a concomitant decrease in p62 in G93A DFX-treated cells, suggesting that the concert of gene mutation and hypoxia stress induced a hyperactivation of autophagy, as confirmed by the detection of vacuoles using immunofluorescence analysis. 

In recent years, many molecules able to modulate the autophagy process have been tested in order to prevent mSOD1-induced motor neuron death [8,45,50]. 

The neuroprotective effect of PACAP has been previously evaluated in mSOD1 motor neurons under exposure to different insults including serum deprivation as well as glutamate excitotoxicity [35]. For the first time, we demonstrated the beneficial effect of PACAP on these cells following hypoxic stress. In this work, we showed that exogenous PACAP administration resulted in a significant reduction of apoptotic death rate concomitant to decreased hSOD1 levels in G93A cells exposed to DFX, suggesting that the downregulation of this gene may also be part of the molecular mechanism underlying PACAP’s effect to rescue motor neurons. Here, we suggested that the PACAP effect was associated with the modulation of autophagic events in motor neurons. Indeed, we observed that PACAP reduced the accumulation of LC3II and increased p62 substrate in G93A cells after DFX exposure, suggesting that neuropeptide modulated hypoxia-induced autophagy dysregulation following SOD1 mutation. These results were confirmed through immunofluorescence analysis showing that PACAP reduced autophagic vacuole formation in DFX treated G93A cells.

Numerous studies have demonstrated that PACAP’s neuroprotective effects were mediated through the activation of the MAPK/ERK pathways. In particular, in our recent work we demonstrated that PACAP treatment was able to prevent serum deprivation-induced G93A cell death by activating this signaling cascade [38]. Therefore, we assessed whether PACAP modulated autophagy dysregulation in these cells following hypoxia exposure through MAPK/ERK signaling activation. Our data demonstrated that PACAP induced ERK phosphorylation in both the WT and G93A DFX-exposed cells. ERK expression was reduced by co-treatment with H89, which is an upstream inhibitor of the MAPK/ERK signaling cascade. 

To verify whether PACAP’s modulatory effect on autophagy was due to ERK phosphorylation, we analyzed LC3I-II expression levels in the presence of H89. The results showed that PACAP-induced LC3II downregulation was reversed by H89 treatment. This suggests that the PACAP effect on autophagy dysregulation could be mediated by MAPK/ERK pathway activation.

The hybrid neuroblastoma-spinal cord (NSC) cell line represents a largely used in vitro model to study motor neurons in an immortalized system. These cells constitutively express many phenotypic characteristics of primary motor neurons, such as the generation of action potentials, neurofilament triplet proteins expression, synthesis/storage of acetylcholine and expression of a receptor for the neuromuscular junction-specific basal lamina adhesion molecule, known as S-laminin [51,52]. As previously demonstrated [53], these cells are sensible to glutamate insult by suggesting their use for cell toxicity studies. Furthermore, these cells can be stably transfected with a plasmid expressing genes bearing SOD1 mutation. In particular, the cells used in the present paper stably expressed G93A SOD1, mimicking a frequent mutation of a familial form of ALS. Data translatability should be evaluated in an in vivo model of ALS represented by mSOD1 mice to correlate these results to other factors such as animal survival and motricity.

## 4. Materials and Methods

### 4.1. Cell Culture

Mouse motor neuron-like hybrid NSC-34 cell line [51] was stably transfected with the pTet-ON plasmid (Clontech, Palo Alto, CA, USA), coding for the reverse tetracycline-controlled transactivator, used to construct inducible cell lines expressing the cDNAs encoding human wild-type-SOD1 (WT) or human SOD1 mutant G93A (G93A). Cells were transfected with human wild type SOD1 (WT) or mSOD1 G93A expressing vector, as previously described [39]. Cells were grown in a mixture of 1:1 Dulbecco’s modified Eagle’s medium (DMEM) and Ham’s F-12K Nutrient Medium (Sigma-Aldrich, St. Louis, MO, USA) supplemented with 15 mM HEPES (Sigma-Aldrich, St. Louis, MO, USA), 10% fetal bovine serum (FBS; Invitrogen, Carlsbad, CA, USA), 100 U/mL penicillin and 100-μg/mL streptomycin (Sigma-Aldrich, St. Louis, MO, USA), and incubated at 37 °C in 5% CO_2_. The expression of hSOD1 was induced by adding 2 µg/mL doxycycline (doxy; Sigma-Aldrich, St. Louis, MO, USA) to the culture medium for the last 24 h of culture. 

### 4.2. Treatments

In each experiment, the cells were exposed to 100 µM desferrioxamine mesylate salt (DFX) (Sigma-Aldrich, St. Louis, MO, USA), a hypoxia-mimetic agent, alone or in combination with 100 nM PACAP38 (Sigma-Aldrich, St. Louis, MO, USA) or 10 µM PAC1 inhibitor, PACAP 6-38 for 24 h. The inhibition of the cAMP-dependent PKA pathway was induced by incubating cells with 50 µM of the specific inhibitor, H89, for 30 min. 

### 4.3. Cell Viability Assay

The number of viable cells was estimated using cell proliferation kit I (MTT) following the manufacturer’s instructions (Roche Diagnostics, Monza, Italy), as previously described by D’Amico et al. [53]. Cells were seeded into 96-well plates at a density of 1 × 10^4^ cells/well in 100 μL of culture medium for one day. After 24 h of induction with doxycycline (Doxy, 2 µg/mL), the cells were subjected to DFX treatment alone or in combination with 100 nM of PACAP-38 (DFX + PACAP) or 10 µM of PACAP 6-38 for 24 h (DFX + PACAP 6-38). The day after the treatments, 0.5 mg/mL of 3-[4–dimethylthiazol-2-yl]-2,5-diphenyltetrazolium bromide (MTT) (Sigma-Aldrich, St. Louis, MO, USA) was added to each well and incubated for 4 h at 37 °C. The reaction was stopped by adding 100 μL of solubilization solution, then formazan formed by the cleavage of the yellow tetrazolium salt MTT was measured spectrophotometrically at 550–600 nm using a microplate reader (BioRad Laboratories, Segrate, MI, Italy). Six replicate wells were used for each group. Controls included untreated cells, whereas the medium alone was used as a blank.

### 4.4. Fluorescence Microscopic Analysis of Cell Death 

Cells were seeded into 12-well plates at a density of 4 × 10^3^ cells/well in 500 μL of culture medium. After 24 h of induction with doxycycline (Doxy, 2 µg/mL), the cells were exposed to 100 µM desferrioxamine mesylate salt (DFX) alone or in combination with 100 nM of PACAP-38 (DFX + PACAP) or 10 µM of PACAP 6-38 for 24 h (DFX + PACAP 6-38). The typical morphological features of apoptotic degeneration were analyzed using confocal microscopy with the nuclear dye Hoechst 33342 [54]. The cells were fixed with a solution of methanol/acetic acid (3:1 *v/v*) for 30 min, washed three times in PBS and incubated for 15 min at 37 °C with 0.4 μg/mL Hoechst 33342 dye. After being rinsed in water, the cells were visualized for the determination of nuclear chromatin morphology with the use of confocal laser scanning microscopy (CLSM; Zeiss LSM700). Each scanning was individually digitized by a high sensitivity photomultiplier tube (PMT) using the following acquisition setup: Gain master: 776; digital offset: −202; digital gain: 1.0. All acquisitions were performed with the ZEN-2010 software. The apoptotic cells were recognized based on nuclear condensation and/or fragmented chromatin. Each condition was reproduced in three dishes per experiment. Both the apoptotic and the normal cells were counted from three fields per dish in a fixed pattern.

### 4.5. Western Blot Analysis

The proteins were extracted from the total cell lysates with a buffer containing 20 mM Tris (pH 7.4), 2 mM EDTA, 0.5 mM EGTA; 50 mM mercaptoethanol, 0.32 mM sucrose supplemented with phosphatase and protease inhibitor cocktails (Roche Diagnostics, Monza, Italy). Subsequently, the protein samples were homogenated by using a Teflon-glass homogenizer and then sonicated twice for 20 s using an ultrasonic probe, followed by centrifugation at 10,000× *g* for 10 min at 4 °C. The protein concentrations were determined by the Quant-iT Protein Assay Kit (Invitrogen, Carlsbad, CA, USA) as previously described by Maugeri et al. [55]. The sample proteins (35 μg) were diluted in 2× Laemmli buffer (Invitrogen, Carlsbad, CA, USA), heated at 70 °C for 10 min and then separated on a Biorad Criterion XT 4–15% Bis-tris gel (BIO-RAD) by electrophoresis and then transferred to a nitrocellulose membrane (BIO-RAD). The blots were blocked using the Odyssey Blocking Buffer (Li-Cor Biosciences, Nebraska, USA) and probed with appropriate antibodies: mouse anti-LC3 (cat. no. sc-398822, Santa Cruz Biotechnology), mouse anti-p62/SQSTM1 (cat. no. H00008878-M01, Abnova), mouse anti-total ERK 1/2 (MK1) (cat. no. sc-135900, Santa Cruz Biotechnology), mouse anti phospho-ERK1/2 (Thr 202/Tyr 204) (cat. no. sc-136521, Santa Cruz Biotechnology), rabbit anti-superoxide dismutase 1 (cat. no. ab79390, Abcam) and rabbit anti-β-tubulin (cat. no. sc-9104, Santa Cruz Biotechnology). 

The secondary antibody goat anti-rabbit IRDye 800CW (cat #926-32211; Li-Cor Biosciences) and goat anti-mouse IRDye 680CW (cat #926-68020D, Li-Cor Biosciences) were used respectively at 1:20,000 and 1:30,000. The blots were scanned with an Odyssey Infrared Imaging System (Odyssey). The densitometric analyses of the Western blot signals were performed at non-saturating exposures and analyzed using the ImageJ software (NIH, Bethesda, MD; available at http://rsb.info.nih.gov/ij/index.html). Values were normalized to β-tubulin used as loading control.

### 4.6. Immunofluorescence Detection of LC3 Protein

NSC-34 cells expressing wild-type SOD1 (WT) or SOD1 G93A (G93A) were cultured in 100 µM desferrioxamine mesylate salt (DFX) alone or in combination with 100 nM of PACAP-38 (DFX + PACAP) on glass cover slips and fixed in 4% paraformaldehyde in PBS (15 min at room temperature), permeabilized with 0.2% Triton X-100 and blocked with 0.1% BSA in PBS for 1 h at room temperature. Subsequently, the samples were probed with the LC3II (1:100, sc-398822) primary antibody overnight and then incubated with the Alexa Fluor 594 goat anti-mouse antibody (green fluorescence) for 1 h at room temperature and shielded from light. DAPI (diamidino-2-phenylindole) was used to stain the nuclei (#940110 Vector Laboratories). 

The images were taken with a confocal laser scanning microscope (CLSM) (Zeiss LSM700). The experiments were repeated at least three times to confirm results [56].

### 4.7. Statistical Analysis 

Data were represented as the mean ± standard error (SEM). A one-way analysis of variance (ANOVA) was used to compare the differences between the groups, and the statistical significance was assessed by the Tukey–Kramer post hoc test. The level of significance for all the statistical tests was set at *p* ≤ 0.05.

## 5. Conclusions

**Figure 8:**
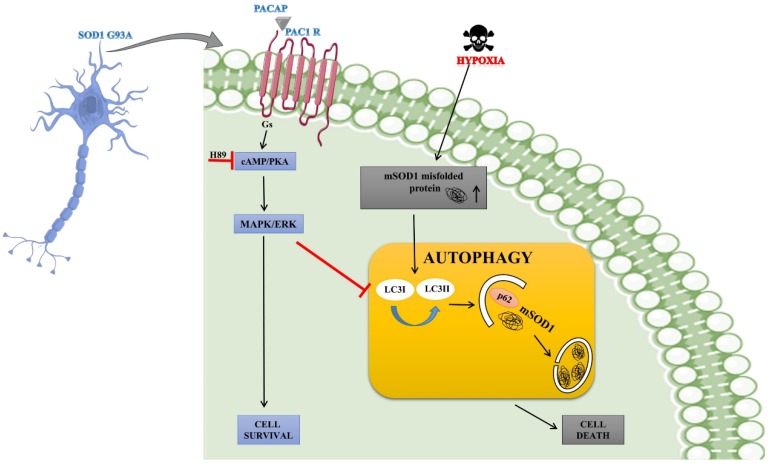
PACAP’s modulatory effect on the autophagy process in SOD1 G93A cells exposed to hypoxia. Hypoxia increased the misfolded mSOD1 proteins causing autophagy dysregulation and cell death. By binding to the PAC1 receptor, PACAP stimulates cAMP production triggering different downstream signaling cascades, including the MAPK/ERK pathway. PACAP-induced ERK phosphorylation regulates the autophagy hyperactivation in the mSOD1 G93A cells leading to their survival.

In conclusion, this study suggests that PACAP has a neuroprotective effect on mSOD1 motor neurons by inhibiting DFX-induced autophagy through MAPK/ERK induction. Overall, our results explain a possible mechanism underlying PACAP neuroprotection in mSOD1 motor neurons, presenting a new perspective to ALS therapy. Figure 8 illustrates our hypothesis regarding PACAP’s modulatory effect on autophagy in SOD1 G93A cells exposed to hypoxia.
